# Transcriptome Analysis Provides Insights into the Safe Overwintering of Local Peach Flower Buds

**DOI:** 10.3390/cimb46120831

**Published:** 2024-12-09

**Authors:** Ruxuan Niu, Yongjuan Cheng, Falin Wang, Yiwen Zhang, Chenbing Wang

**Affiliations:** Institute of Fruit and Floriculture Research, Gansu Academy of Agricultural Sciences, Anning, Lanzhou 730070, China; rxniu@gsagr.cn (R.N.); chengyj@gsagr.ac.cn (Y.C.); wfl@gsagr.ac.cn (F.W.); zhangyw@gsagr.cn (Y.Z.)

**Keywords:** dormancy, low temperature and cold resistance, peach flower buds, antioxidant enzymes, transcriptome

## Abstract

During the dormant period of peach trees in winter, flower buds exhibit weak cold resistance and are susceptible to freezing at low temperatures. Understanding the physiological and molecular mechanisms underlying the response of local peach buds to low-temperature adversity is crucial for ensuring normal flowering, fruiting, and yield. In this study, the experimental materials included the conventional cultivar ‘Xia cui’ (XC) and the cold-resistant local resources ‘Ding jiaba’ (DJB) peach buds. The antioxidant enzyme activity, levels of malondialdehyde (MDA), proline (Pro), and hydrogen peroxide content (H_2_O_2_) were determined in peach buds at different dormancy periods. Transcriptome sequencing was performed at three dormancy stages: the dormancy entry stage (FD), deep dormancy release stage (MD), and dormancy release stage (RD). Additionally, transcriptome sequencing was conducted to analyze gene expression profiles during these stages. Our findings revealed that compared with XC cultivars, DJB peach buds exhibited decreased MDA and H_2_O_2_ contents but increased superoxide dismutase (SOD), peroxidase (POD), and catalase (CAT) activities as well as Pro content during the dormancy period. These findings suggest that cold-resistant cultivars possess significantly stronger antioxidant capacity than conventional cultivars under low-temperature stress. A total of 10,168 differential genes were annotated through transcriptome sequencing. Among them, 4975 were up-regulated while 5193 were down-regulated. The differentially expressed genes associated with low-temperature response in peach buds are primarily enriched in plant hormone signal transduction pathway and phenylpropane synthesis pathway. Key differentially expressed genes related to cold resistance include *ARF2*, *GH3*, and *SAPK2*, and differentially expressed transcription factors mainly belong to the *AP2/ERF-ERF*, *bHLH*, and *C2H2* families. This study provides a theoretical foundation for understanding the key genes involved.

## 1. Introduction

The peach tree (*Prunus persica* L.) is extensively cultivated worldwide and holds significant importance in terms of food and economic value [1]. It exhibits a wide distribution in northwest China and enjoys popularity among people. However, its growth faces numerous challenges due to various environmental factors. In recent years, the occurrence of extreme weather events, particularly severe winters, has had a profound impact on open-air cultivation of peach trees. This leads to bud freezing during dormancy, subsequently affecting fruit setting rate, development, and yield [2].

Dormancy is a biological adaptation mechanism developed by plants to cope with seasonal environmental changes [3]. It can be categorized into heteromorphic dormancy, endogenous dormancy, and ecological dormancy based on inducing factors [4]. Fruit tree dormancy primarily refers to growth stagnation caused by internal structures (such as tissues or organs) themselves, for instance, due to low-temperature requirements or photoperiodic effects. In the winter dormant period, the flower buds of cultivated peach trees exhibit weak resistance to low temperatures and are prone to freezing. When frozen, these buds may turn black, leading to no germination or a reduced germination rate in the spring. Eventually, the buds dry up, wither, and fall off. However, the resistance of flower buds from local resources in Gansu province is significantly higher than that of cultivated species. Owing to its central location within the mainland, the western region of China is characterized by arid climates and low winter temperatures, rendering it susceptible to frost damage. Notably, specific zones in Gansu Province endure severe frost conditions, a result of the interplay between the region’s topography and climatic patterns. Gansu is nestled in the northwest of China, at the confluence of the Loess Plateau, the Qinghai-Tibet Plateau, and the Inner Mongolia Plateau, boasting a complex and varied terrain and a spectrum of climatic conditions [5]. The majority of the province is under the temperate continental climate, featuring four distinct seasons. Precipitation varies significantly across the region, with an annual decrease from 805.7 mm in the southeast to a mere 36.8 mm in the northwest. Most areas receive an average annual precipitation ranging from 40 to 750 mm, and arid and semi-arid zones constitute 75% of the total provincial area [6]. Gansu experiences a substantial annual and diurnal temperature range, and except for the southern part of the central region, the annual sunshine duration surpasses 2400 h, with the Hexi Corridor’s areas often exceeding 3200 h. This temperature variation fosters the accumulation of sugar in peaches, markedly improving the fruit’s quality [5]. During winter, Gansu is susceptible to cold air influxes, resulting in generally low temperatures, and the fruit industry is particularly vulnerable to frost damage. Certain economically significant fruit trees, such as peach trees, are prone to frost damage in winter, which can lead to diminished fruit quality and reduced yields. Given that flowering and fruit production depend entirely on normal bud development, it is essential to study their cold resistance. The cold resistance of flower buds has been documented in previous studies [7,8].

In the study of plant cold resistance, osmoregulatory substances, malondialdehyde, and antioxidant enzymes are employed as evaluation indicators for assessing cold resistance [9]. Reactive oxygen species (ROS) play a pivotal role in plant adaptation to abiotic stress. They are an inevitable byproduct of aerobic metabolism and participate in the regulation of multiple metabolic pathways involved in plant stress response; however, they can also exert detrimental effects during this process [10]. Under low-temperature stress conditions, an imbalance occurs leading to excessive production of reactive oxygen species such as superoxide anion radical (O_2_^−^), hydrogen peroxide (H_2_O_2_), hydroxyl radical (OH), and singlet oxygen (^1^O_2_). Consequently, elevated intracellular ROS levels result in oxidative damage to cells and membrane systems [11]. Nevertheless, most cellular compartments within plant cells contain abundant antioxidant enzymes including superoxide dismutase (SOD), catalase (CAT), and peroxidase (POD) [12]. Crucial modulator substances like proline acid (Pro) can reduce cellulite permeation potential, rapidly enhance the ability to counteract excess ROS accumulation, and effectively eliminate surplus ROS molecules [13].

During hypothermia, plants have developed several mechanisms to minimize the potential damage caused by low temperatures. With the rapid advancement of transcriptomics and increasingly sophisticated technology, transcriptomics technology has been extensively employed in recent years to investigate the mechanism of plant cold response. From these research findings, crucial key genes for cold resistance and the associated pathway mechanism have been identified [14]. The molecular basis of cold acclimation and the acquisition of frost resistance in plants has been extensively examined. In order to adapt to cold stress, gene expression and metabolism undergo changes during cold acclimation. When plants are exposed to cold stress, there is an increase in cytoplasmic Ca^2+^ concentration which induces structural alterations mediated by Ca^2+^-binding proteins. This activation leads to the activation of other binding proteins and transmission of the low-temperature signal to downstream target genes, resulting in transcriptional and metabolic modifications as a response to low-temperature stress [15].

Cold stress induces Ca^2+^ signaling via multiple pathways in plants. Plant species exhibit a wide range of calcium receptors, such as calmodulin (CaM) and calmodulin-like proteins (CML), calcine-dependent protein kinases (CDPKs), calcine-dependent protein kinases (CCaMK), calmodulin-binding transcriptional activators (CAMTA), calcineurin B-like proteins (CBL), and calcineurin interacting protein kinases (CIPKs) [16]. Genetic analysis has demonstrated that CDPKs act as positive regulators [17], whereas CaM3 functions as a negative regulator for both plant gene expression and cold tolerance [18]. Additionally, CBL interacts with the CIPK family to regulate the transmission of Ca^2+^ signals [19]. CAMTA3 binds to a cis-acting element known as the CG-1 element or vCGCGb in its promoter region, thereby facilitating positive regulation of the cold response gene *CBF2*/*DREB1C* [20]. Among various cold signal transduction pathways, the CBF/DREB1-dependent pathway is extensively characterized as the principal regulatory pathway [21], primarily composed of *ICE*, *CBF*, and *COR* genes involved in inducing the expression of cold-responsive genes [22]. Previous studies have demonstrated that *ICE*, *CBF*/*DREB1*, and *COR* genes play crucial roles in plant responses to low temperatures. A comprehensive understanding of the molecular mechanisms underlying plant cold tolerance will expedite breeding efforts aimed at enhancing traits related to cold tolerance and provide potential candidate genes for further investigations [23].

In conclusion, the activity of antioxidant enzymes and cold-responsive genes in plants plays a crucial role in enhancing their resistance to low temperatures. Therefore, this experiment establishes a physiological foundation for transcriptomic sequencing by evaluating the physiological indicators of dormant peach flower buds. Additionally, it analyzes differentially expressed genes under low-temperature stress conditions, identifies potential candidate genes related to cold resistance in peach flower buds, elucidates the adaptability of these buds to withstand cold stress effectively, and provides a theoretical basis for cultivating peaches with improved tolerance against low temperatures.

## 2. Material and Methods

### 2.1. Plant Materials

The two peach varieties employed in this study included the conventional cultivar ‘Xia cui’ (XC) and the cold-resistant local resources ‘Ding jiaba’ (DJB). They were cultivated within a dedicated peach resource nursery situated in the Institute of Forestry, Fruit and Flower of Gansu Academy of Agricultural Sciences. The cultivation area is situated within a temperate semi-arid climatic region, characterized by coordinates of longitude 103 degrees 41 min and latitude 36 degrees 6 min, at an elevation of 1530 m. The region experiences an average annual temperature of 8.9 °C, boasting a frost-free period spanning 172 days. During winter, the average low-temperature dips to −13 °C, with the record low reaching an extreme of −21.7 °C. The soil composition within this open field nursery comprised sandy loam with moderate fertility levels. Row spacing was established at 1.5 m × 5 m intervals to ensure optimal growth conditions for each plant species under investigation. The sampling time of this experiment is from November 2022 to March 2023, and samples are collected on the 1st and 15th of each month. Six plants were selected from each variety, and 3–5 annual branches of similar thickness, free from disease and insect pests, and exhibiting good lignification were chosen from the four cardinal directions (southeast, northwest), respectively. Flower buds were carefully plucked from the fruit-bearing branches, immediately frozen in liquid nitrogen, and stored at −80 °C in a refrigerator for subsequent determination of various physiological indices and transcriptomic sequencing. Each index was replicated three times to ensure biological reproducibility.

### 2.2. Measurement of Physiological Indexes

The level of lipid peroxidation, an indicator of oxidative stress, was determined by measuring the malondialdehyde (MDA) content using the thiobarbituric acid (TBA) reaction [24]. Pro concentrations, which signal osmotic adjustments, were assessed with the sulfosalicylic acid ninhydrin method [25]. H_2_O_2_ levels were measured through spectrophotometry [26]. Enzymatic assays for peroxidase (POD), SOD, and CAT were conducted using kits provided by Suzhou Ke Ming Biotechnology Co., Ltd., Suzhou, China.

### 2.3. Transcriptome Sequencing

Beijing Biomec Biotechnology Co., Ltd. (Beijing, China) conducted transcriptome sequencing of peach flower buds using the NEBNext^®^ Ultra™ RNA Library Preparation Kit (NEB Corporation, Fond du Lac, WI, USA) to construct sequencing libraries. The PCR products were purified with the AMPure XP system and library quality was assessed using Agilent Bioanalyzer 2100. TruSeq PE Cluster Kit V3-CBOTt-HS (Illumina, San Diego, CA, USA) on the Illumina HiSeq platform was employed for encoding and clustering appropriate libraries followed by sequencing. Gene expression levels were evaluated by calculating the FPKM value of each gene, and differential gene expression analysis was performed using the DESeq2 method with screening thresholds set at FDR ≤ 0.05 and Log2FC ≥ 2 to identify differentially expressed genes.

### 2.4. Statistical Analysis

The experimental data underwent statistical analysis using SPSS 22.0 software, employing single-factor Duncan detection (*p* < 0.05). Additionally, Origin 2021 software was employed for data visualization. A *p*-value of less than 0.05 was deemed statistically significant. All data points are elucidated as mean ± standard error.

## 3. Results

### 3.1. Changes of Physiological and Biochemical Indexes in Different Dormant Periods

**Figure 1:**
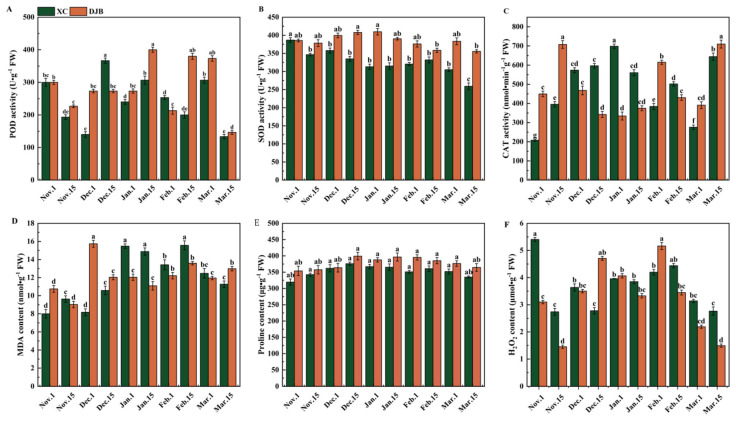
Effects of antioxidant enzyme activity, MDA, Proline, and H_2_O_2_ content in XC and DJB peach flower buds at different dormant stages. Note: (**A**–**C**) POD, SOD, and CAT enzyme activities of peach flower buds at different dormant stages. (**D**) MDA content of peach flower buds at different dormant stages. (**E**) The Proline content of peach flower buds in different dormant stages. (**F**) H_2_O_2_ content in peach flower buds at different dormant stages. Letters a–f in the figure indicate data significance, (*p* < 0.05).

To further investigate the impact of low temperatures on the antioxidant capacity of peach buds, we quantified the levels of antioxidant enzymes in peach flower buds. The results revealed that under low-temperature treatment, the activities of CAT, POD, and SOD in DJB flower buds were higher compared to the control group (XC). Additionally, on 15 November, 15 January, 15 February, 1 March, and 15 March, CAT, POD, and SOD activities in DJB flower buds were significantly higher than those in XC (Figure 1A–C). In conclusion, exposure to low temperatures during the dormancy period enhanced the peach’s ability to eliminate reactive oxygen species while reducing lipid peroxidation and dehydration characteristics of cell membranes. Consequently, this protective mechanism safeguards flower buds against cold damage.

The objective of this study was to investigate the impact of the dormancy period on the osmotic pressure of flower bud cell membranes. Measurements of MDA, Pro, and H_2_O_2_ levels in peach flower buds were conducted on specific dates: 1 November, 15 November, 1 December, 1 January, 15 January, 1 February, 15 February, 1 March, and 15 March. The results revealed an initial increase followed by a subsequent decrease in MDA, Pro, and H_2_O_2_ contents in flower buds with prolonged dormancy periods. Notably, DJB flower buds exhibited lower MDA content compared to XC flower buds. Furthermore, there was a significant decrease specifically observed on 15 March for DJB flowers (Figure 1D). Additionally, DJB flower buds displayed higher proline content than XC flowers; elevated proline levels are known to confer protection against cold stress to plant cells (Figure 1E). On 1 November, 15 November, and 15 January, there was a significant reduction in H_2_O_2_ content for DJB flower buds. Conversely, XC flower buds generally exhibited higher H_2_O_2_ levels compared to DJB (Figure 1F).

### 3.2. Transcriptome Data Statistics

Transcriptomic sequencing was conducted on peach flower buds at the dormancy entry stage (15 November), deep dormancy release stage (15 January), and dormancy release stage (15 March). The reference genome used was Prunus_persica. Chinese_Cling_v1.0.genome.fa. Each sequencing sample yielded an average of 43,866,027 read segments, presenting a GC content of 45.53% and a Q30 value of 94.88%. Moreover, each sample achieved an average of 655 million nucleotide sequences, with an alignment efficiency of 92.62%. (Table S1).

### 3.3. Transcriptome DEGs Enrichment Analysis

Based on physiological changes observed in flower buds during different stages of dormancy progression—entering dormancy (FD), deep dormancy release stage (MD), and final dormancy release stage (RD)—this study conducted a transcriptome analysis to identify differential gene expression patterns among dormant-stage samples. A total of 10,168 differentially expressed genes were identified in the transcriptome analysis. Among them, 588, 5090, and 4490 genes were found to be differentially expressed in X-FD vs. D-FD, X-MD vs. D-MD, and X-RD vs. D-RD comparisons, respectively. Additionally, we observed up-regulation of 290, 1708, and 2977 genes while down-regulation of 298, 3382, and 1513 genes (Table 1).

**Figure 2:**
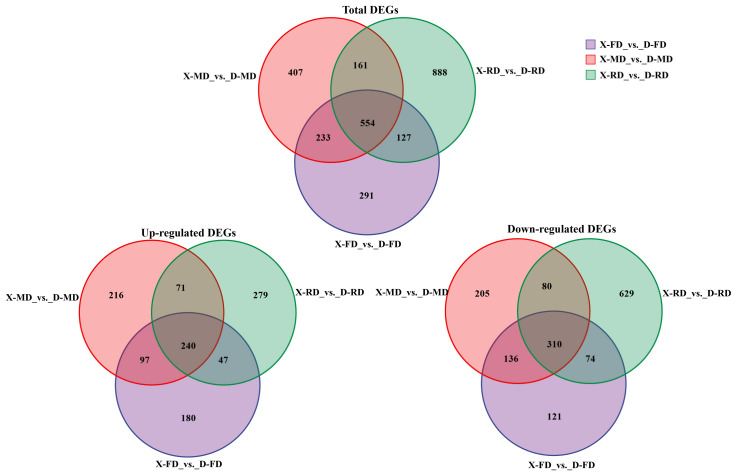
The total number of DEGs in peach flower buds in different dormant periods, and the Venn diagram of up-regulated and down-regulated DEGs. Note: X: XC; D: DJB. FD: Dormancy entry period (15 November). MD: Period of deep dormancy (15 January). RD: Period of dormancy release (15 March). Same as below.

The expression levels of 228 genes were upregulated, while 184 genes were downregulated in the comparison between the X-FD and D-FD stages. In the comparison between X-MD and D-MD stages, there were 1641 specifically upregulated differentially expressed genes (DEGs) and 3288 specifically downregulated DEGs. Furthermore, in the comparison between X-RD and D-RD stages, there were 2880 specifically upregulated DEGs and 1441 specifically downregulated DEGs (Figure 2). Generally, a higher number of DEGs was observed in flower buds at the MD stage compared to those at the RD and FD stages. Additionally, dormant flower buds exhibited the lowest number of DEGs with no significant difference observed between up- and down-regulated DGEs.

### 3.4. Transcriptome GO Enrichment Analysis

To further investigate the enrichment of specific functional categories in peach bud transcriptomes during different dormant stages, we assessed the main biological functions of DEGs through gene ontology-based analysis and GO category enrichment using a threshold value (*p* ≤ 0.05). The enriched categories can be classified into three groups: biological process (BP), cell component (CC), and molecular function (MF).

**Figure 3:**
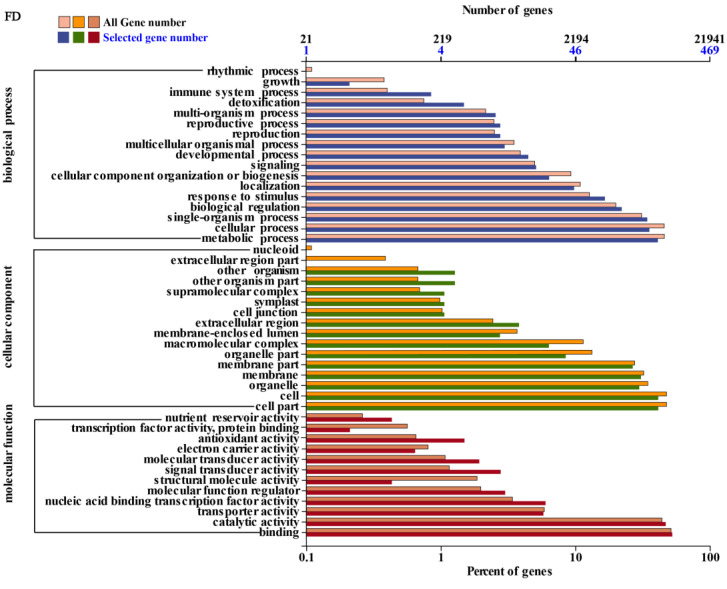
Classification of GO annotation of differentially expressed genes in flower buds at dormant entry (FD) stage. Note: The ordinate is the GO classification, the lower edge of the abscissa is the percentage of the number of genes, and the upper edge is the number of genes. This figure shows the gene enrichment of each secondary function of GO in the background of differentially expressed genes and all genes, reflecting the status of each secondary function in the two backgrounds, and the secondary functions with obvious proportional differences indicate that the enrichment trend of differentially expressed genes is different from that of all genes, and we can focus on analyzing whether this function is related to the difference, the same below.

**Figure 4:**
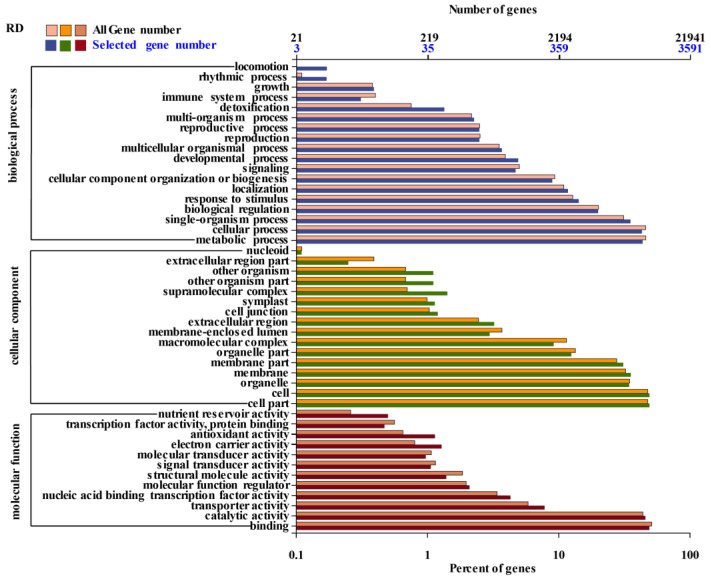
Classification chart of GO annotation of differentially expressed genes in flower buds during dormancy release (RD) stage.

During FD and RD, DEGs primarily participate in metabolic processes, cellular regulation, regulation of individual organisms, and biological processes, as illustrated in Figure 3 and Figure 4. Concerning cell component categories, the majority of DEGs are predominantly associated with organelle components, membrane components, membranes themselves, cells, and cellular components. In terms of molecular function, the majority of DEGs mainly engage in binding activity and catalytic activity.

**Figure 5:**
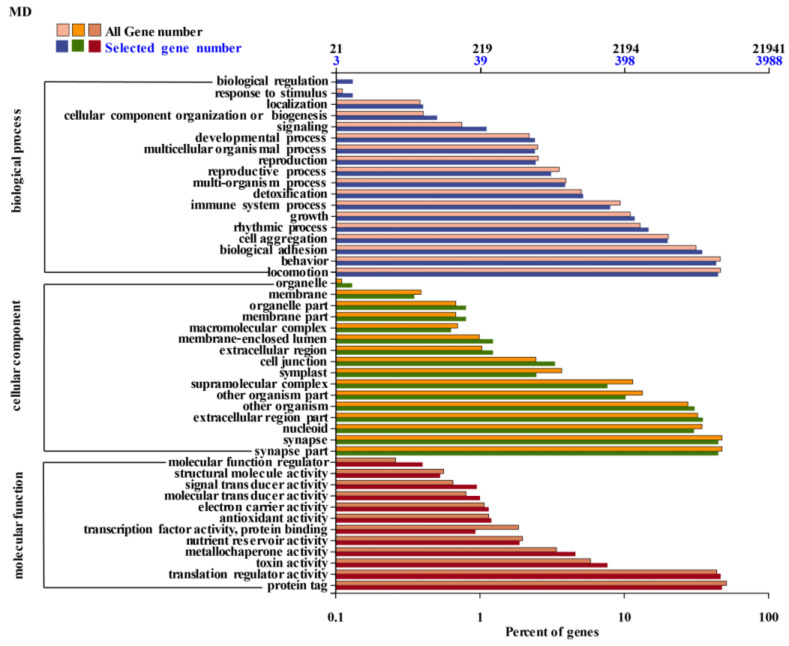
Classification of GO annotation of differentially expressed genes in flower buds during deep dormancy (MD).

During the MD period, a significant proportion of DEGs were implicated in locomotion, rhythmic processes, and cell aggregation (Figure 5). In terms of cellular components, DEGs primarily participated in synaptic parts, synapses, nucleoids, other organisms, and extracellular regions. Regarding molecular functions, the majority of DEGs were predominantly associated with protein tagging and translation regulation activities. The transcriptome analysis revealed similar transcriptional dynamics in the FD and RD stages, as evidenced by the GO enrichment analysis conducted at different dormancy stages. Notably, the largest number of genes exhibited differential expression in the X-RD_ vs. _D-RD difference group.

### 3.5. KEGG Enrichment Analysis of Transcriptome

The flower buds of XC and DJB in FD, MD, and RD periods were subjected to enrichment analysis and annotation using KEGG. A comparative transcriptomic analysis was performed between the overwintering flower buds of cold-resistant and conventional cultivars to gain a comprehensive understanding of their differences.

**Figure 6:**
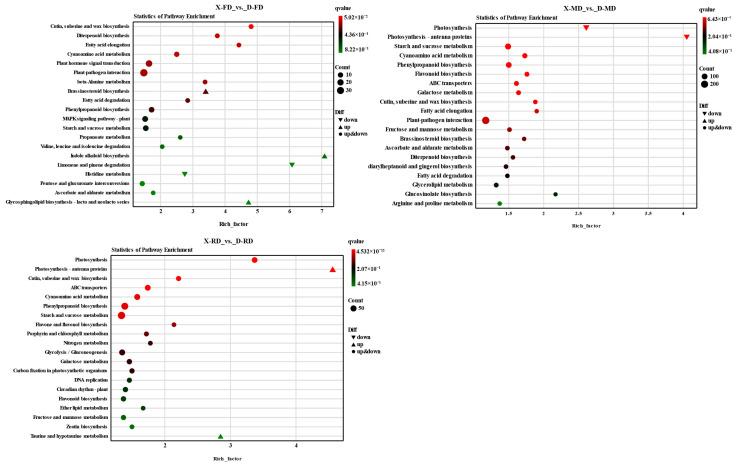
Bubble map of KEGG enrichment of differentially expressed genes. Note: The abscissa is the Gene Ratio, which is the ratio of the gene of interest in the annotation to the number of all differentially expressed genes, and the ordinate is for each pathway entry. The size of the dot represents the number of differentially expressed genes annotated in the pathway, and the color of the dot represents the q value of the hypergeometric test.

The KEGG pathway analysis was conducted to identify the significantly enriched pathways of DEGs (Figure 6). In X-FD_ vs. _D-FD, the enrichment analysis revealed significant associations with biosynthesis processes including cutin, suberine, and wax biosynthesis; diterpenoid biosynthesis; fatty acid elongation, cyanoamino acid metabolism; plant hormone signal transduction; plant-pathogen interaction; and beta-alanine metabolism. In X-MD_ vs. _D-MD, the main pathways were related to photosynthesis, photosynthesis-antenna proteins, starch and sucrose metabolism, cyanoamino acid metabolism, phenylpropanoid biosynthesis, flavonoid biosynthesis, ABC transporters, galactose metabolism as well as Cutin, suberine and wax biosynthesis in response to plant-pathogen interactions.

Enrichment analysis of KEGG pathways in X-RD_ vs. _D-RD revealed significant involvement in photosynthesis, photosynthesis-antenna proteins, cutin, suberine and wax biosynthesis, ABC transporters, cyanoamino acid metabolism, phenylpropanoid biosynthesis, and starch and sucrose metabolism. Notably, the most significantly enriched pathways across all three comparison groups were plant-pathogen interaction as well as cutin, suberine, and wax biosynthesis, cyanoamino acid metabolism, phenylpropanoid biosynthesis, along with starch and sucrose metabolism.

The above results demonstrate that DJB flower buds were mainly involved in sucrose metabolism, phenylpropanoid biosynthesis, plant hormone signal transduction, flavonoid biosynthesis, photosynthesis-antenna proteins, and other pathways. The enrichment of DEGs in these pathways gave plants greater tolerance to low temperatures.

### 3.6. The Visualization of Pathways

**Figure 7:**
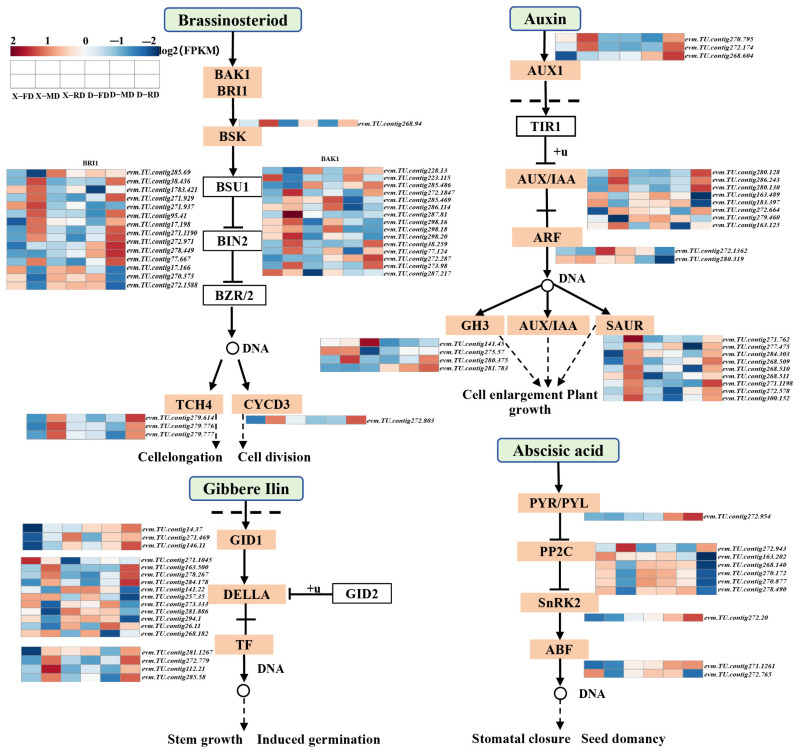
Expression of genes related to phytohormone signaling pathways.

The hormonal pathways of IAA, ABA, GA, and BR signaling were investigated in this study (Figure 7). Our findings revealed that the D-RD treatment led to up-regulation of the *AUX1* gene in the IAA signal transduction pathway. Notably, we observed a significant increase in expression levels of the gamma-aminobutyric acid (GAT-1) *GABA* (*evm.TU.contig268.60*) gene in DJB compared to XC. Conversely, Auxin response factor *ARF2* (*evm*.*TU*.*contig280*.*319*) exhibited a significant down-regulation in DJB.

The expression level of the *GH3* gene varied across different time periods. Specifically, under X-RD treatment, the expression level of indole-3-acetate amide synthetase *GH3*.6 (*evm.TU.contig141.45*) was significantly up-regulated, while DJB exhibited a significant down-regulation compared to XC in all three periods. Within the ABA signaling pathway, *PYL2* and *SnRK2* genes (recombinant serine/threonine protein kinase SAPK2) were consistently down-regulated during all three phases of XC but showed an up-regulation during D-FD, D-MD, and D-RD phases. Apart from *evm*.*TU*.*contig272*.*943* and evm.TU.contig163.202 members of the PP2C family, other PP2C family members demonstrated a down-regulation in X-MD and D-RD periods but an up-regulation in the remaining four periods. Additionally, Ferredoxin (FD) gene (*evm*.*TU*.*contig271*.*1261*) belonging to ABF (ABA reaction element binding protein) family displayed an up-regulation during FD, MD, and RD stages of DJB cold-resistant cultures.

In the GA signaling pathway, the expression of the *GID1* gene in gibberellin insensitive dwarf is up-regulated during D-MD and D-RD stages, while the carboxylesterase *CXE18* (*evm*.*TU*.*contig14*.*37*) exhibits higher expression levels in DJB compared to XC. Within the BR signaling pathway, *evm.TU.contig272.287* of BAK1 family members and *evm*.*TU*.*contig272*.*971* and *evm*.*TU*.*contig278*.*449* of BRI1 family members are down-regulated in XC but up-regulated in DJB. These findings suggest that peach flower buds interact with plant hormones during dormancy, influencing the expression of crucial genes and ultimately impacting various aspects of plant growth (such as stomatal regulation, seed dormancy, and senescence), as well as responses to stress.

**Figure 8:**
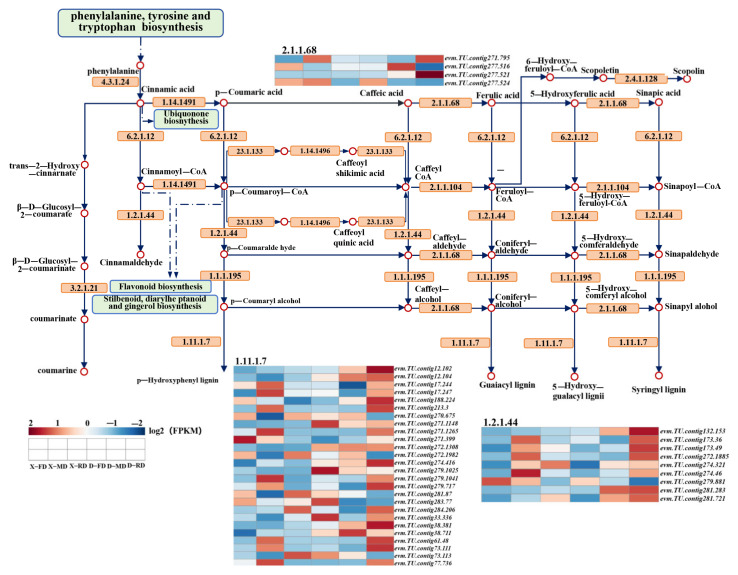
Expression of genes related to phenylpropanoid biosynthesis pathway.

The expression patterns of DEGs in the phenylpropane biosynthesis pathway were analyzed under various treatments, as shown in Figure 8. It was observed that during the RD stage, the DJB gene exhibited up-regulation compared to XC, indicating that low temperature can induce the expression of *PRX* (peroxidase), *COMT* (catechol-O-methyltransferase), and *TKPR1* (pyranone reductase) genes. Furthermore, within the same time frame, members of the PRX family including *PRX A2* (*evm.TU.contig12*.*102*), *PRX42* (*evm*.*TU*.*contig38*.*711*), *PRX19* (*evm*.*TU*.*contig38*.*381*), and *PRX20* (evm.TU.contig274.416) showed down-regulation in XC but significant up-regulation in DJB.*COMT* gene (*evm*.*TU*.*contig277*.*521*) and *TKPR1* gene (*evm*.*TU*.*contig132*.*153*) were also down-regulated in XC but exhibited a significant increase in DJB.

### 3.7. The Analysis of Trends in Gene Co-Expression

**Figure 9:**
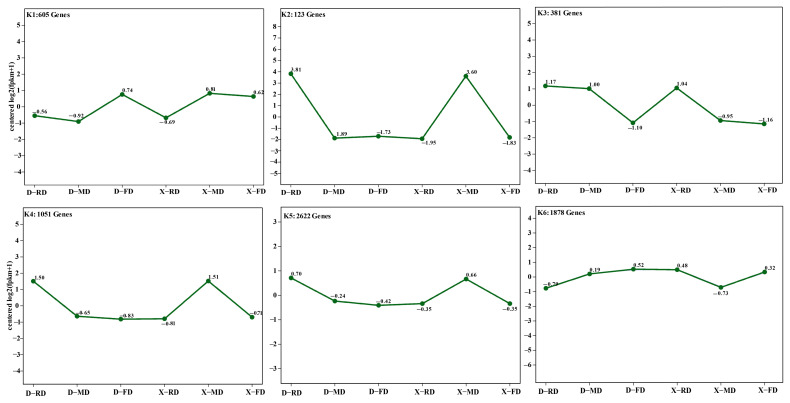
Gene co-expression clusters.

According to the chronological order, genes exhibiting similar expression trends in the samples were clustered and their expression patterns were analyzed. The K-means clustering method was employed for this purpose. To investigate the overwintering mechanism of peach flower buds during the dormancy period, transcriptomes of conventional cultivar XC and cold-resistant cultivar DJB were sequenced at three time points: early dormancy (FD), mid-dormancy (MD), and late dormancy (RD). By performing differential analysis between these time points (X-FD_ vs. _D-FD, X-MD_ vs. _D-MD, X-RD_ vs. _D-RD) and conducting trend analysis using K-means clustering, a total of 6660 genes were classified into six distinct groups denoted as K1 to K6 (Figure 9).

Compared to the conventional variety (XC), the cold-resistant variety (DJB) exhibited higher expression of K3 genes (381 Genes). However, the expression of the other five genes varied significantly among different flower bud varieties at different stages. During the FD period, a slightly higher expression trend was observed for Genes in K1 (605 Genes), K2 (123 Genes), K3 (381 Genes), and K6 (1878 Genes) in DJB flower buds compared to XC flower buds, but no statistically significant difference was found. The expression levels of genes in the K2 and K4 were significantly upregulated in DJB flower buds compared to XC during the RD stage, while a downregulation trend was observed for K6. During the MD stage, the expression trends of genes in K3, K4, and K5 in DJB flower buds were significantly higher than those in XC flower buds.

### 3.8. The Prediction of Transcription Factors

**Figure 10:**
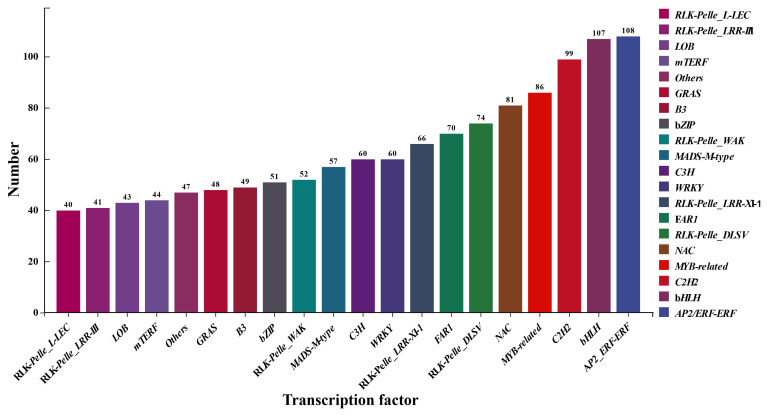
Transcription factor prediction.

To further investigate the cold tolerance mechanism of peach flower buds, We conducted comprehensive predictions on two varieties of peach buds. The prediction results showed that the top three transcription factor families with the most annotations were *AP2/ERF-ERF*, *bHLH*, and *C2H2*, respectively (Figure 10). The AP2/ERF-ERF family is a crucial transcriptional regulatory family involved in various developmental processes and environmental stress responses across different organisms. Additionally, the bHLH family is widely recognized as a significant transcriptional regulator in plant growth, development, and environmental response. The *C2H2* family has also been demonstrated to have a key role in numerous organisms. These findings provide valuable insights for exploring the molecular mechanism underlying cold resistance.

### 3.9. The Correlation Heat Map

**Figure 11:**
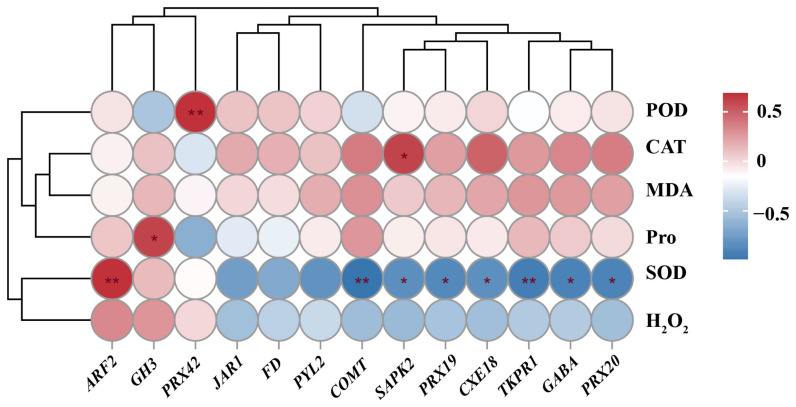
Correlation analysis of FPKM values of differential genes and physiological indexes. * indicates that the *p*-value is less than 0.05 and the correlation is significant. ** Indicates that the *p*-value is less than 0.01 and the correlation is extremely significant.

In order to gain a deeper understanding of the correlation between physiological indicators and differential genes, a correlation analysis was performed between differentially expressed gene (FPKM) expression levels and physiological markers (Figure 11). The results demonstrated a significant negative correlation between the FPKM values of *COMT* and *TKPR* genes and SOD activity (*p* < 0.01), with correlation coefficients of −0.97 and −0.93, respectively. Additionally, *SAPK2*, *PRX9*, *CEX8*, *GABA*, and *PRX20* exhibited a significant negative correlation with SOD activity (*p* < 0.05), with correlation coefficients of −0.82, −0.86, −0.81, −0.89, and −0.89, respectively. Additionally, the correlation coefficients between *ARF2* and SOD and *PRX42* and POD were 0.91 and 0.98, respectively, and the correlation was extremely significant (*p* < 0.01). *GH3* was positively correlated with Pro and *SAPK2* was significantly positively correlated with CAT (*p* < 0.05), with coefficients of 0.84 and 0.88, respectively. However, *JAR1*, *FD*, antioxidant oxidase activity, MAD, and H_2_O_2_ content in flower buds did not exhibit any significant correlations (*p* > 005). These findings suggest that *ARF2*, *GH3*, and *SAPK2* may play more crucial roles than other genes in conferring cold tolerance to flower buds.

## 4. Discussion

### 4.1. Relationship Between Physiological and Biochemical Indexes and Cold Resistance of Peach

Bud dormancy is a critical mechanism employed by perennial fruit trees to withstand adverse natural conditions and successfully complete their life cycle. Several factors influence dormancy, including tree species and variety [27], external factors such as light, temperature, and moisture [28], internal factors like hormones, sugars, and enzymes [29], dormancy-related genes *PHY*, *DAM*, etc. [30,31], as well as epigenetic regulatory mechanisms [32,33]. Through long-term evolution, higher plants acquire advantageous biological traits that enable them to adapt to extreme environmental conditions and seasonal changes. Thus, bud dormancy serves as a responsive mechanism towards adverse environments (e.g., low temperature).

Low-temperature stress exerts a profound detrimental impact on the physiological processes of plants, resulting in peach bud abortion, reduced yield, and economic losses. This poses a significant challenge to the sustainable development of China’s peach industry [34]. In recent years, there has been considerable emphasis on comprehending the response to low-temperature stress and breeding cold-tolerant plant varieties [35]. During low-temperature stress, plants generate free radicals that accumulate and disrupt the balance between reactive oxygen species production and removal within cells. Consequently, this triggers peroxidation of membrane lipids, protein polymerization and denaturation on membranes, increased membrane permeability, ultimately leading to membrane damage [36]. Plant cells possess an array of self-protective antioxidant enzyme mechanisms such as MDA, SOD, POD, and CAT. These enzymes regulate cell membrane permeability while maintaining normal metabolism and safeguarding structural-functional stability [37].

It was observed that the activities of POD, SOD, and CAT in plants increased under low-temperature stress, exhibiting an initial rise followed by a decline with prolonged exposure to low temperatures. During the early stage of low-temperature stress, cellular oxidation levels escalated, prompting a upregulation of enzyme activities as a protective response against cell damage. However, as cells approached their tolerance limit to low temperatures during later stages, continued destruction ensued leading to cell death. Consequently, enzyme activity diminished [38]. Starzenska et al. [39] conducted measurements on SOD activity in flower buds of various broccoli cultivars during short-term storage and discovered a significant increase in SOD activity under low-temperature conditions. Bezirganoglu et al. [40] investigated the response patterns of pea genotypes with varying cold resistance under cold stress and found that cold acclimation enhanced both SOD and CAT activities, which were positively correlated with cold resistance levels. Genotypes displaying strong cold resistance exhibited high CAT activity while those with poor cold resistance demonstrated lower enzyme activity.

In this study, the antioxidant metabolic physiological indices of flower buds from the conventional cultivar ‘XC’ and the cold-resistant cultivar ‘DJB’ were investigated during natural dormancy. Significantly different activities of CAT, SOD, and POD were observed between the two cultivars, with higher levels found in DJB compared to XC. These findings suggest a positive correlation between CAT, SOD, and POD activity and cold resistance in peach flower buds. Previous studies by Sagisaka et al.’s [41] demonstrated that perennials such as poplars, metasequoia, ginkgo biloba, Daphne, and winter wheat accumulate high levels of proline under overwintering conditions. Golubev et al.’s observation [42] also reported an increase in free proline content at low temperatures positively associated with enhanced cold resistance in apricot flower buds. Consistent with these results and Sagisaka et al.’s observations [41], our study revealed a fluctuating pattern of Pro content during the dormancy period of peach flower buds; initially decreasing followed by an increase before declining again. Moreover, DJB exhibited higher proline content than the XC cultivar. Numerous studies have indicated that MDA content increases in plants exposed to low temperatures. Our findings demonstrate lower MDA content in cold-resistant DJB flower buds compared to conventional XC cultivars suggesting a correlation between MDA accumulation and cold resistance in peach flower buds.

### 4.2. Plant Hormone Signal Transduction Pathway Cold Resistance Gene

Plant hormones play a pivotal role in enhancing the cold resistance of plants, and the intricate interplay between these hormones is indispensable for plants to rapidly and effectively respond to both biotic and abiotic stressors. Numerous studies have demonstrated that hormones such as ABA, GA, Auxin, and BR regulate various aspects of plant growth and development while also playing a crucial role in mediating plant signal transduction during stress responses [43]. RNASeq technology, renowned for its remarkable precision and cost-effectiveness, has emerged as an invaluable tool for transcriptome analysis in plant functional genomics research focused on unraveling the intricate correlations underlying inverse tolerance [44].

The transcriptome data of this experiment showed that low temperature could affect the expression of genes in the plant hormone signal transduction pathway. Among the three families of auxin early response (*GH3*, AUX/*IAA*, and *SAUR*), the positive regulator *SAUR* exhibits the highest rate of change [45]. Emerging evidence indicate the involvement of auxin in the regulation of plant growth and development under low-temperature stresses. *GH3* genes encode auxin-conjugating enzymes and modulate endogenous levels of active auxin through negative feedback regulation. In rice, overexpression of *OsGH3*-*2* increased cold tolerance and reduced free IAA content, alleviated oxidative damage, and decreased membrane permeability [46]. Specifically, *SAUR* is predominantly down-regulated in XC but mostly up-regulated in DJB flower buds. Additionally, homologous genes such as *GH3* and *ARF* are up-regulated in XC. Brassinosteroids (BRs) are another class of phytohormones that play a key role in plant development and defense. BRs regulate the expression of several COR genes and CBF regulon, thereby controlling the freezing tolerance [47]. In terms of the BR signaling pathway, different varieties exhibit distinct changes in both positive and negative regulatory factors during the dormant period under low-temperature conditions. Several reports suggest that the BRs are involved during the cold stress response. BRI serves as a positive regulatory factor within the BR signaling pathway, with studies indicating that Arabidopsis bri1 mutants display enhanced tolerance to low temperatures [48].

Notably, no significant differences were observed in the number of up-regulated and down-regulated DELLA homologous genes within the gibberellin (GA) pathway; however, differentially expressed *GID1* homologous positive regulators were predominantly up-regulated in cold-resistant varieties. Therefore, it can be speculated that gibberellins play a positive regulatory role in peach bud dormancy and its response to low temperatures. In Arabidopsis thaliana, GA binds to *GID1* and activates interaction between *GID1* and DELLA protein which leads to tighter binding between DELLA protein and *GID2* ultimately facilitating entry into the 26S degradation pathway [49].

Abscisic acid (ABA) is a bonafide stress hormone that plays key functions during stress [50]. Several reports suggest that ABA concentration elevates against stress in conjunction with the regulation of several genes. During stress conditions, carotenoids are converted into bioactive ABA by the de novo biosynthesis pathway. ABA biosynthesis is increased during cold stress, which improves a plant’s ability to resist during unfavorable cold conditions. ABA biosynthesis and catabolic genes display their regulation in organ organ-dependent manner during cold stress. Plants subjected to cold stress induce a number of ABA-responsive genes [51]. The ABF family plays a crucial role in the regulation of hormone signaling. In the ABA signaling pathway, *PP2C* activity is inhibited by *PYR/PYL*, leading to the activation of downstream *SnRK2* and subsequent initiation of ABF expression. It has been demonstrated that ABF members are responsive to environmental factors, such as adversity, during ABA signal transduction. Furthermore, they exhibit strong adaptability to low temperatures and other stressful conditions in plants [52]. The findings from this study revealed that *PYL2* and *SAPK2* were down-regulated at all three stages of XC but up-regulated at D-FD, D-MD, and D-RD stages. Additionally, they were up-regulated at FD, MD, and RD stages in DJB cold-resistant cultures. SnRK2 protein kinase *SAPK2* acts as a positive regulator in the abscisic acid signaling pathway. Apart from phosphorylating transcription factors to regulate gene expression, *SAPK2* can directly activate metabolism-related proteins and modulate plant stress resistance. Overexpression of *SnRK2* gene and wheat *SnRK24* gene in Arabidopsis thaliana significantly enhances its tolerance to low temperature [53].

### 4.3. Relationship Between Phenylpropane Synthesis Pathway and Cold Resistance

Phenylpropane compounds are secondary metabolites synthesized by plants in response to stress and specific environmental conditions, such as variations in light intensity, temperature, and nutrient availability [54]. These compounds are widely distributed throughout the plant kingdom and encompass a diverse range of benzene derivatives including total flavonoids, flavonols, coumarins, lignin, anthocyanins, and tannins. With thousands of chemical structures available, phenylpropane compounds play a pivotal role in plant growth and development. They are closely associated with plant defense against toxins, mechanisms for stress protection, regulation of flower and fruit pigmentation, modulation of cell composition as well as signal transduction molecules. Therefore, they play a crucial role in enabling plants to effectively cope with abiotic stresses [55].

Low-temperature conditions can enhance the phenylalanine synthesis pathway [56]. Based on the transcriptome data from this study, catalase *PRX* is identified as a key gene in the phenylpropane metabolic pathway of cold-tolerant cultivars during dormancy. PRX69 responds to low temperatures, helping plants improve and better adapt to their environment [57]. *PRX* plays a crucial role in ROS production for plant stress responses. For instance, Arabidopsis *PRX33* and *PRX34* are primarily involved in defense response through ROS generation [58]. Receptor kinases regulate enzyme activity associated with endogenous ROS production via phosphorylation and dephosphorylation, thereby promoting intracellular ROS accumulation and initiating defense responses. As these enzymes exhibit differential responses to various types of stress, plants can discriminate between different stress signals by detecting distinct types of ROS produced under specific stresses and respond accordingly [59].

Furthermore, phenylpropanoid compounds have demonstrated remarkable efficacy in response to various environmental stresses, including suboptimal temperatures. They significantly contribute to enhancing plant resilience against adverse conditions such as low temperatures and play a crucial role in maintaining regular physiological processes by actively regulating water balance and ion homeostasis. In summary, as secondary metabolites, phenylpropane compounds perform multifaceted essential functions during plants’ adaptation to diverse stressors. A comprehensive understanding of their metabolic mechanisms will expedite the exploration of invaluable insights for enhancing plant adaptability and improving agricultural cultivars while providing guidance for future scientific investigations within related fields.

### 4.4. Relationship Between Transcription Factors and Cold Resistance

Transcription factors (TFs) are a class of proteins that specifically bind to cis-elements in the promoter regions located upstream of the start codon of a target gene to regulate transcription [60]. TFs are key regulators of gene expression at the transcriptional level, helping plants respond to environmental signals. The *AP2/ERF-ERF* TFs have been reported to play a regulatory role in plants in response to abiotic stress [61]. *AP2/ERF-ERF* TFs were highly expressed in peach with strong cold resistance under low-temperature stress [62]. The *bHLH* family is among the largest groups of TFs in plants and plays a crucial role in various response pathways to abiotic stresses, including water scarcity, high salinity, low temperatures, and nutrient deficiencies [60]. Since the identification of the *bHLH* family in plants, numerous studies in model species have investigated the functions and regulatory mechanisms of *bHLH* family members in the context of abiotic stress responses. In recent years, *bHLH* TFs have been extensively researched for their role in regulating abiotic stress responses and resistance in horticultural crops. Li et al. [63] conducted an analysis of peach transcriptomics and metabolomics under cold stress, revealing that the expression levels of *AP2/ERF-ERF* and *bHLH* were elevated in cold-tolerant peach varieties compared to cold-sensitive ones. This suggests that *AP2/ERF-ERF* and *bHLH* may play a potential role in regulating genes associated with cold tolerance.

The *C2H2* transcription factors are one of the most significant families of transcriptional regulators in plants. They are key components in the regulation of plant growth, development, hormone responses, and tolerance to both biotic and abiotic stresses [64]. Quantitative analysis of their expression patterns has shown that *MdC2H2* genes are rapidly induced upon exposure to abiotic stresses such as low-temperature [65]. These findings imply that *AP2/ERF-ERF*, *bHLH*, and *C2H2* transcription factors may be closely linked to the cold resistance of peach flower buds. These results will aid in uncovering the molecular mechanisms underlying cold tolerance development and will provide a scientific foundation for the future improvement of fruit tree varieties.

## 5. Conclusions

In summary, the physiological indices indicated that flower buds exhibiting strong cold resistance displayed robust antioxidant enzyme activity. Transcriptome data revealed that low temperatures during the dormant period influenced the synthesis and metabolism of secondary metabolites, as well as the interaction between hormones, by regulating key genes involved in phenylpropane biosynthesis, plant hormone signal transduction, and photosynthesis. Consequently, these factors affected peach growth and development along with its tolerance to cold stress. *GH3*, *ARF2*, and *SAPK2* were identified as potential candidate genes associated with peach bud’s cold resistance.

## Figures and Tables

**Table 1:** Statistics on the number of differential genes.

DEG Set	DEG Number	Up-Regulated	Down-Regulated
X-FD vs. D-FD	588	290	298
X-MD vs. D-MD	5090	1708	3382
X-RD vs. D-RD	4490	2977	1513

Note: X: XC; D: DJB. FD: Dormancy entry period (15 November). MD: Period of deep dormancy (15 January). RD: Period of dormancy release (15 March).

## Data Availability

Data is contained within the article or Supplementary Materials: The data presented in this study are available in insert article.

## Supplementary Materials

The following supporting information can be downloaded at: https://www.mdpi.com/article/10.3390/cimb46120831/s1, Table S1. Statistics of transcriptome sequencing data of peach flower buds in the dormancy entry period, deep dormancy periodand dormancy end period.
