# Supplementary material for: Transcriptome Analysis Provides Insights into the Safe Overwintering of Local Peach Flower Buds
Source: Curr Issues Mol Biol. 2024 Dec 9;46(12):13903–21. doi: 10.3390/cimb46120831 (PMC11727394; doi:10.3390/cimb46120831)
Supplement: Supplementary file 1 [file cimb-46-00831-s001.zip › cimb-3316469-supplementary.pdf]

Table S1. Statistics of transcriptome sequencing data of peach flower buds in the dormancy entry period, deep dormancy periodand dormancy end period.

variety	Period of dormancy	Total Reads	Clean bases	Mapped Reads	GC Content	%≥Q30	Mapped Ratio%
XC	Nov.15	42,271,095	6,319,229,488	39,097,196	45.69	95.50	92.49
	Jan.15	46,896,674	7,010,950,832	42,451,004	45.14	94.49	90.44
	Mar.15	41,289,232	6,172,579,052	39,033,583	45.51	94.62	94.54
DJB	Nov.15	49,223,075	6,264,174,634	46,362,402	45.71	95.53	94.06
	Jan.15	41,604,271	7,362,671,068	38,600,446	45.63	94.55	92.75
	Mar.15	41,911,821	6,223,204,732	38,339,656	45.52	94.56	91.42

Note: (1) variety: Test material varieties; (2) Period of dormancy: dormant period; (3) Total Reads: total number of Clean Data, calculated by single end; (4) Clean bases: total number of Clean Data bases; (5) Mapped Reads: number of Reads compared to the reference genome; (6) GC content: Clean DataGC content, i. e. percentage of G and C bases in Clean Data; (7) Q30%: percentage of bases with Clean Data mass value greater than or equal to 30; (8) Mapped Ratio: The percentage of Reads in the Clean Reads aligned to the reference genome.
